# Model-based algorithms to ascertain smoking in administrative health data: a registry-based validation study

**DOI:** 10.23889/ijpds.v9i5.2846

**Published:** 2024-09-18

**Authors:** Md Ashiqul Haque, Nathan C Nickel, Maxime Turgeon, Lisa M Lix

**Affiliations:** 1 Department of Community Health Sciences, University of Manitoba; 2 Department of Statistics, University of Manitoba

## Objectives

We developed a machine-learning model-based algorithm (MBA) for smoking in Administrative Health Data (AHD). The validity of this MBA was compared to a rule-based algorithm (RBA).

## Approaches

The study included adults (≥18 years) from a clinical registry containing self-reported current smoking from 2017 to 2020 in Manitoba, Canada. Clinical data were linked to up to five years of hospitalization, physician billing claims, and prescription medication records. The RBA was based on diagnosis codes for tobacco use and nicotine dependence medication. MBAs, constructed using random forest (RF) models, included these indicators in addition to comorbid condition diagnoses and sociodemographic factors. Sensitivity, specificity, positive and negative predictive values (PPV, NPV), and 95% confidence intervals (CIs) were estimated.

## Results

The cohort comprised 24,718 individuals (88.6% female); prevalence of current smokers was 10.0%. The RBA had sensitivity of 27.3% (95% CI: 24.2-30.7), specificity of 96.6% (95% CI: 96.1-97.0), and PPV of 47.2% (95% CI: 42.9-51.5). The MBA had sensitivity of 68.6% (95% CI: 65.1-71.9), specificity of 76.3% (95% CI: 75.2-77.3), and PPV of 24.3% (95% CI: 23.2-25.6). NPV was high irrespective of algorithms. Stratified analyses revealed similar estimates for males and females, and the number of years of AHD did not affect the MBA results.

## Conclusions

An RF-based MBA for smoking ascertainment in linked AHD sources improved sensitivity compared to the RBA. However, the RBA excelled in specificity and PPV.

## Implication

Balancing accurate smoker identification with the risk of false positives is crucial when choosing an algorithm to ascertain current smokers using AHD.

